# The shared mother-child epigenetic signature of neglect is related to maternal adverse events

**DOI:** 10.3389/fphys.2022.966740

**Published:** 2022-08-24

**Authors:** Inmaculada León, Silvia Herrero Roldán, María José Rodrigo, Maykel López Rodríguez, Jonah Fisher, Colter Mitchell, Agustín Lage-Castellanos

**Affiliations:** ^1^ Instituto Universitario de Neurociencia, Universidad de La Laguna, San Cristóbal de la Laguna, Spain; ^2^ Facultad de Psicología, Universidad de La Laguna, San Cristóbal de la Laguna, Spain; ^3^ Department of Pathology and Experimental Medicine, University of California, Los Angeles (UCLA), Los Angeles, CA, United States; ^4^ Institute for Social Research, University of Michigan, Ann Abor, MI, United States; ^5^ Department of NeuroInformatics, Cuban Center for Neuroscience, Havana, Cuba; ^6^ Department of Cognitive Neuroscience, Faculty of Psychology and Neuroscience, Maastricht University, Maastricht, Netherlands

**Keywords:** DNA methylation, intergenerational effects, mother-child epigenetic mark, maternal life adversity, neglectful mothering

## Abstract

Studies of DNA methylation have revealed the biological mechanisms by which life adversity confers risk for later physical and mental health problems. What remains unknown is the “biologically embedding” of maternal adverse experiences resulting in maladaptive parenting and whether these epigenetic effects are transmitted to the next generation. This study focuses on neglectful mothering indexed by a severe disregard for the basic and psychological needs of the child. Using the Illumina Human Methylation EPIC BeadChip in saliva samples, we identified genes with differentially methylated regions (DMRs) in those mothers with (*n* = 51), versus those without (*n* = 87), neglectful behavior that present similar DMRs patterns in their children being neglected versus non-neglected (*n* = 40 vs. 75). Mothers reported the emotional intensity of adverse life events. After covariate adjustment and multiple testing corrections, we identified 69 DMRs in the mother epigenome and 42 DMRs in the child epigenome that were simultaneously above the α = 0.01 threshold. The common set of nine DMRs contained genes related to childhood adversity, neonatal and infant diabetes, child neurobehavioral development and other health problems such as obesity, hypertension, cancer, posttraumatic stress, and the Alzheimer’s disease; four of the genes were associated with maternal life adversity. Identifying a shared epigenetic signature of neglect linked to maternal life adversity is an essential step in breaking the intergenerational transmission of one of the most common forms of childhood maltreatment.

## 1 Introduction

Growing evidence has shown that exposure to childhood maltreatment and adverse life events can disrupt healthy development by increasing the risk to physical and mental health (Felitti, 2009; Jaffee, 2017; Nelson et al., 2020). In the specific case of childhood maltreatment (for example, abuse or neglect), its life-long consequences include a greater risk of violence and delinquency, as well as adult depression and attempted suicide (Gilbert et al., 2009), persistent smoking and alcoholism (Dube et al., 2006; Kisely et al., 2020), or obesity (Danese and Tan, 2014). DNA methylation (DNAm), as a critical mechanism in epigenetic regulation, has been proposed as one of the molecular processes to explain how life adversity experiences confer risk for later physical and mental health problems (Nestler et al., 2009; Zhang and Meaney, 2010). Different studies have confirmed this relationship in individual child and adult samples (see Cecil et al., 2016; Parade et al., 2021, for a review). However, much less is known about the biological embedding of these adverse experiences resulting in maladaptive parenting. An interesting case is mothers who show extreme disregard and insensitivity toward their children (Petersen et al., 2014). These mothers have been frequently exposed to maltreatment in their infancy, a history of adverse life events, early childbearing of many children, and intimate partner violence, and mostly had a low educational level and lived in families facing substantial economic hardship (Bartlett et al., 2014; Mulder et al., 2018; Herrero-Roldán et al., 2021a).

The current study focuses on this type of neglectful caregiving, the most common and severe form of maltreatment, which consists of the mother’s failure to provide the child with food, clothing, shelter, medical care, supervision, or emotional support (Stoltenborgh et al., 2013; Petersen et al., 2014; US Department of Health and Human Services, 2019). Neglect entails cumulative risk for infant mental health and behavioral problems (Petersen et al., 2014), and carries out neurobiological alterations across the life span (see review by Teicher and Samson, 2016). Epigenetics could likely be acting as one of the mechanisms that may explain how environmental risk factors throughout development can contribute biologically to explaining the neglectful phenotype. Some recent results may go in that direction, showing a higher epigenetic age acceleration (EAA) for mothers who neglect their child, using the DNAm PhenoAge clock (Herrero-Roldán et al., 2021b). EAA was even higher for these mothers with risk factors such as low education or living with an unstable partner.

A biological substantiation of maternal life adversity resulting in poor caregiving takes us one step further, warranty searching for possible shared epigenetic signatures between mothers with neglect behavior and their children. To our knowledge, no study has addressed whether a particular epigenetic pattern of neglect characterizes both the mothers and their children who suffer from neglect. Besides, most of the methylation studies based on analyses of mother-child data are limited by candidate-gene approaches, which only look for specific genes and CpG sites, precluding a broader understanding of methylation patterns across the genome (Kertes et al., 2016; Parade et al., 2021).

The first aim of this study was to identify genes with differentially methylated regions (DMRs) in mothers with neglectful behavior that present similar methylation patterns in their neglected children. The second aim was to examine the potential association between the shared DMRs and the maternal emotional intensity of adverse life events. Little is known about how epigenetic alterations in human mothers can be found in the next generation. Finding commonalities between mothers and children may support overlapping DNAm patterns since both may have experienced adverse childhood events and share an immediate family context. Furthermore, both are exposed to common physical environmental factors known to affect epigenetic changes during prenatal and peri-postpartum periods (Perera and Herbstman, 2011; Lim et al., 2017). This is the case of the diet and, to some extent, the same toxic stressors, such as the same bacteria and pollution (Lim et al., 2017). Finally, they also may share some intergenerational epigenetic modifications, as animal research suggests (Whitelaw and Whitelaw, 2008; Aiken et al., 2016). We examined DNA methylation (DNAm) profile differences in a sample of 51 mothers in the neglect group and 87 mothers in the non-neglectful control group and 40 children in the neglect group and 75 children in the control group, through saliva samples. We used saliva as a suitable and less invasive mean for sample collection showing comparable methylation profiles and similar sensitivity to interindividual methylation differences than blood (Murata et al., 2019). Methylation in saliva appeared more similar to patterns from several brain tissues examined overall than methylation in blood (Smith et al., 2015). We relied on epigenome-wide association studies (EWASs) that have used the Illumina Human Methylation EPIC BeadChip array to identify particularities in the DNAm of individuals with different phenotypes or diseases (Jaffe et al., 2012; Pidsley et al., 2016). Identifying the shared epigenetic marker of neglect would help improve our understanding of dysfunctional parenting and the potential factors involved in its intergenerational transmission and its critical and long-lasting consequences.

The second related aim was to test whether the overlapping pattern of mother-child differential methylation obtained in our first aim was related to the maternal exposure to adverse life events. This measure refers to the emotional intensity of adverse events that happen throughout life which confers a risk of chronic stress. Proven the methylation-adversity relationship is a more specific test to claim the biological embedding of the mother’s life adversity in the shared mother-child epigenetic modifications. It is likely that at least part of the overlapping mother-child methylation would be related to genes associated with early adversity and social stress, such as *PM20D1* (Suderman et al., 2014; Benson et al., 2019); *RUFY1* (Martins et al., 2021); *SLC17A3* (Suderman et al., 2014); *MRPL28* (Wiegand et al., 2021) and *AURKC* (Martins et al., 2021); see other potential genes in the reviews by Cecil et al. (2016) and Parade et al. (2021) in both children and adults. However, confirmation of mother-child DNAm common profiles in epigenome-wide studies is still scant and weak. Maternal post-traumatic stress disorder, following the experience of sexual violence/torture during the Kosovo war, was related to their children’s differential methylation at candidate genes *NR3C1*, *HTR3A,* and *BNDF*; however, no methylation differences reached epigenome-wide corrected significant levels (Hjort et al., 2021).

Our study can generate better evidence on a possible intergenerational epigenetic profile in the neglect phenotype. In neglect dyads, both the mother and child suffer social stress directly and indirectly, potentially increasing the probability of shared DNAm, involving stress-related genes and others. We relied on overlapping mother-child methylated genes in a closely defined sample, not looking at specific genes but using the epigenome-wide array. Furthermore, finding associations between maternal risk factors and overlapping methylated profiles would help explain the intergenerational transmission of neglect.

## 2 Materials and methods

### 2.1 Participants

Two hundred and fifty-five participants: 51 mothers and 40 of their children in the neglect group (NG), and 89 mothers and 75 of their children in the non-neglectful control group (CG), were recruited through the same Municipal Social Services and Primary Health Centers. Two mothers in the control group were removed after the initial quality control. The total number of children did not coincide with the mothers since we could not obtain the salivary sample of 22 children. In another case, the quality of the sample did not reach the level of DNA necessary for subsequent analyses. All mothers gave their written informed consent also for their children following the protocol of the Ethical Committee of Investigation of the Canary Islands University Hospital Complex (code: CHUC_2018_63; date of approval: 14 December 2018), under the Code of Ethics of the World Medical Association (Declaration of Helsinki).

General inclusion criteria for both mother and child groups were being the biological mother of a child under 7 years old who had not been placed in foster care at any point in their history nor been born prematurely or suffered perinatal or postnatal medical complications, according to the pediatricians’ reports. Specific inclusion criteria for a mother in the neglect group were a substantiated case of child neglect registered in the last 12 months by Child Protective Services (CPS) according to the reports of the Social Services and complying with all the indicators of the Maltreatment Classification System (MCS) for severe neglect (Barnett et al., 1993) according to the pediatrician of the Primary Health Center in charge of the case. Only cases with the exclusive presence of child neglect (that is, the mother who commits neglect and the child who exclusively suffers it) were included to avoid the co-occurrence of other types of maltreatment in the child, as pointed out by Kim et al. (2017). The specific inclusion criteria for the control group were being biological mothers of children having negative scores in all the MCS neglect indicators and without CPS or Preventive Services records for the family.

As shows Table 1, mothers in the NG were younger and had more children than mothers in the CG, and the target child had a similar mean age and sex distribution in both groups. Moreover, NG mothers were less likely than mothers in the CG to live in two-parent families and more likely to show a lower educational level and to receive financial assistance than those in the CG, whereas both groups showed a moderate-high percentage of unemployment and a lower percentage of mothers living in rural than in urban areas.

**TABLE 1:** Sociodemographic profile in control and neglect groups.

Mothers	Control group *M* (*SD*) or %	Neglect group *M* (*SD*) or %	*t(136)*/*χ2*
	*n* = 87	*n* = 51	
Current age	33.98 (6.13)	30.67 (7.36)	2.84**
Age at child´s birth	30.94	28.09	2.52*
Number of children	1.66 (0.73)	2.49 (1.29)	−4.25***
Two-parent family %	72	49	6.63**
Level of education %		18.76***
Primary	43	80	
Secondary school	52	18	
> Secondary school	5	2	
Rural areas %	26	43	3.35
Unemployment %	59	71	1.49
Financial assistance %	24	68	24.58***
**Children**	***n* = 75**	***n* = 40**	***t*(113)/*χ*2**
Mean age of target child	3.84 (2.17)	4.06 (2.77)	−0.47
Male %	58	45	1.45

*p < .05; **p < .01; ***p < .001. Note: Group comparisons with mean scores were performed with t statistic, while those with percentage values were performed with Chi-Square (χ2) statistic.

### 2.2 Psychological measure

The Stressful and Risky Events Inventory (SREI) was developed by combining items from other questionnaires (ACE, Dube et al., 2002; ISER, Hidalgo et al., 2005) according to their relevance to our risky population. It comprises 16 self-reported adverse events (e.g., divorce, economic pressure, chronic illness, eviction, unwanted pregnancy) that are likely to happen throughout their lives. Each item was rated on a categorical scale (no/yes occurrence) and its emotional impact on the participant was scored on a 3-point Likert scale (0 = no occurrence; 1 = little impact; 3 = very high impact). The total emotional impact was obtained by a cumulative scoring of the emotional intensity of the adverse events suffered.

### 2.3 Procedure

Social workers reported on the participants’ family characteristics and asked mothers for permission for them and for their children to be contacted. Those mothers who gave permission were informed about the study and the procedure upon their written acceptance. We avoided the use of the term “neglect” in the contact communications. The collaborator visited their homes and collected their responses to the questionnaire and the saliva samples for them and their child. The Real Saliva DNA Sample Collection Kit (Ref. RBMSAL01) was used for mothers, and the pediatric Genotek DNA Sample Collection Kit OC-175 was used for children. Monetary compensation was given to the mothers at the end of the session.

## 3 DNAm assay and methylation analyses

DNA was extracted from the saliva samples at the University Hospital N. S. de Candelaria (Tenerife, Spain), using the extraction Kit (Maxwell^®^ 16 LEV Blood DNA Kit (Cat. #AS1290). Concentration and purity of DNA was assessed by spectrophotometry. Quality assessment of DNA samples was performed with the TapeStation instrument. Library preparation and methylation sequencing were conducted at the University of Michigan Epigenomics Core in Ann Arbor (Michigan, United States). In short, 250 ng of sample DNA was bisulfite converted with Zymo Kits using the manufacturer’s incubation parameters specific for Illumina methylation arrays. The cleaned-up samples were then hybridized using the Illumina Infinium Human Methylation EPIC BeadChip (Illumina Inc., San Diego, United States). Raw red/green IDAT files were read into R using the Ewastools package (Heiss and Just, 2018). Green and red idat pairs were loaded and beta methylation value were computed as M/(U + M+100). RELIC dye bias correction was applied to adjust for performance differences between dye types (Xu et al., 2017). Detection *p* values were calculated. Probes with over average detection *p* > 0.05 were cut (*n* = 52,188). Subsequently, samples with average detection *p* > 0.1 were cut (*n* = 2). Cross reactive probes (Pidsley et al., 2016) were cut (*n* = 41,963). This left us with 253 samples (*n* = 138 mothers, and *n* = 115 children) and 771,785 probes. Snakemake was used to treat bioinformatics workflow.

### 3.1 Statistical analysis

For our first aim, a whole epigenomic search for differentially methylated regions (DMRs) was implemented separately for mother and child datasets. Then, we explored the overlap between mother and children in DMRs when analyzing the effect of neglect vs. control. The relationship between mother and child was explored by correlating the effect of neglect between mother and child datasets at the level of the whole epigenome. This approach has the advantage of being independent of the threshold used for significance. Next, we selected as interesting candidates DMRs that simultaneously survived the significant threshold of *p*-value < 0.01 in both datasets.

For our second aim, we analyzed the relationship between the methylation levels in the set of genes corresponding to the DMRs shared by mother and child and self-reported maternal adverse life events. The data was corrected for possible batch effect arising from the samples run in different plates. The relative proportion of epithelial cells was included in the analysis as a covariate. We did not include the proportion of leukocytes because this measure was highly collinear with the measure of epithelial cells.

#### 3.1.1 Differentially methylated regions identification

The search for differentially methylated regions was implemented with the Bumphunter algorithm (Jaffe et al., 2012). Bumphunter starts by defining CpG clusters of probes that are around the same genomic region. The Single Nucleotide Polymorphisms (SNP) containing CpGs are excluded to reduce the potential effect of genetic variation on the results. Next, a linear model is estimated at each probe and the coefficient of interest is extracted, in our case the coefficient of interest is the effect of neglect. The response variable for this linear model is the methylation level for each probe. The design matrix contains the variable of interest (neglect vs. control dummy coded) and other covariates to be considered (epithelial cells, maternal chronological age and level of education in the mother data, and child age and maternal age at child’s birth in the child data). After fitting the linear model, the profile of the coefficient of interest is chromosomically smoothed across consecutive genomic positions within the same clusters. The statistics for measuring the relevance of each bump is the area of the smoothed coefficient profile above a used defined threshold. The statistical significance of each bump was estimated non-parametrically with bootstrap and the *p-*value associated with each bump was retrieved. A total of 1,000 boostrap samples were used.

To reduce the risk of false discoveries we followed a strategy that automatically corrects for multiple comparisons. The test’s *p*-values between neglect and control mothers and neglected and control children were converted to z-statistics which is more appropriate for computing correlations. Then, we correlated the observed z-statistics in the mother’s dataset with the observed z-statistics in the child’s dataset across the whole epigenome. The parameter settings used in the Bumphunter function are: pickCutoff = TRUE, pickCutoffQ = 0.95, nullMethod = “bootstrap”, B = 1,000, smooth = TRUE, smoothFunction = loessByCluster, useWeights = FALSE, maxGap = 1,000. The intersection of significant DMRs between the two datasets was calculated using a *p*-value threshold of 0.001 in the mothers and the children’s data. The restriction of being significant in both datasets at the same time reduces the risk of false discoveries.

#### 3.1.2 Relationship of the overlapping differentially methylated regions with maternal life adversity

After determining the significant DMRs distinguishing neglect versus control conditions in mothers and children, the relationship between the methylation levels and the mother’s SREI total scores of the emotional intensity of adverse life events, as a covariate of interest, was analyzed. We used Bumphunter under the same design matrix and hyperparameters used in the previous section in this analysis. The only difference was adding the SREI scores in the design matrix and considering them as the target variable. The significant relationships between the covariable of interest and the methylation levels was determined using a *p*-value threshold of 0.001. We also crosschecked with the literature the function of these genes and the evidence of their relation to life adversity.

## 4 Results

### 4.1 Mother-child correlations for differentially methylated regions

**FIGURE 1:**
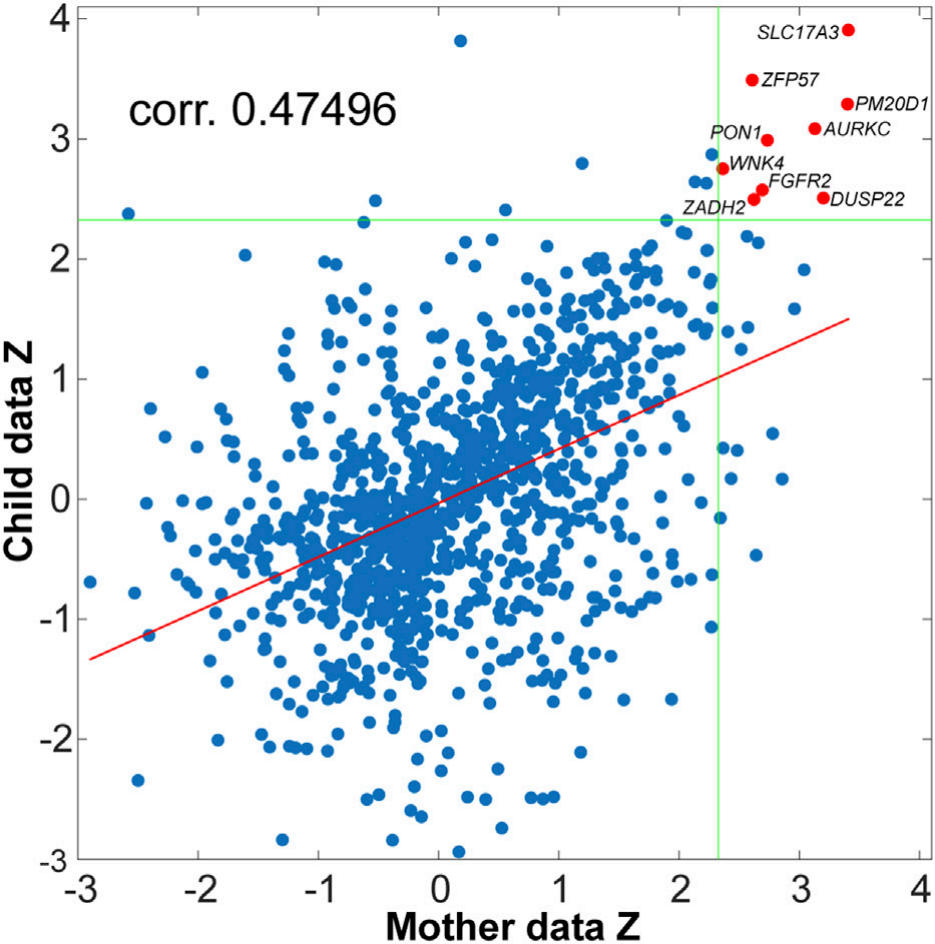
Scatter plot of the *p*-values (converted to *Z*-scale) for the mother and the child data. Each dot in the figure corresponds to the resulting clusters of CpGs showing differences in methylation level between neglect and control, tested for mother and child data separately. The dots in red (two of them indicating two genes) correspond to DMRs where the difference between neglect and control was significant for α = 0.01 in both datasets simultaneously. The Z value denoting a significance threshold of α = 0.01 is marked by the green lines, and the regression line between mother and children Z’s is denoted in red.

For the first aim, we hypothesized that common mother-child genes exist where the effect of neglect can be observed on their methylation levels. We first implemented separated DMR searches in the mother and child datasets using Bumphunter (see Methods) to investigate this possibility. Bumphunter detected 8,671 and 7,167 (bumps) candidate regions as potential DMRs in the mother and child dataset, respectively. A total of 1,193 (bumps) candidate regions were shared between both sets. A scatter plot between the *p*-values (z-transformed) of mother and children candidate regions is presented in Figure 1.

A correlation of 0.47 (*p*-value < 1e-6) was observed between mother and child *z*-statistics (see Methods for the definition of the *z*-statistics). This result shows that being in the neglect group has similar influences on the mothers’ and children’s epigenomes, without depending on a threshold for statistical significance. Under a significance threshold of α = 0.01 (uncorrected) we observed 69 DMRs in the mother’s dataset where neglect and control mothers showed differences in their methylation level. Under the same threshold, in the child dataset we observed 42 DMRs with differences between neglected and control children. Next, we focused our interpretations on those DMRs that were below the α = 0.01 threshold in both datasets (mother and child) simultaneously. This strategy automatically corrects for multiple comparisons due to reducing the probability of observing extreme values under chance in both datasets simultaneously, from 0.01 to 0.01^2 = 1E-4. We observed nine differentially methylated regions common between both datasets (red points in Figure 1).

Table 2 shows the features of the nine differentially methylated regions common to mother and child (13% of the mother dataset and 21.4% of the child dataset). Cluster length varied from 8 to 24 CpGs and significant probes within clusters varied from 7 to 22 (see first and second columns). Methylation N > C Mother/Child indicates the sign of the difference in each dataset: a positive value means that the Neglect group has larger methylation levels than the Control group (see third column). The majority of overlapping sites map to regulatory regions indicating promoters, as defined by genomic location (e.g.,Transcription start sites (TSS) and flanking (TSS), histone marks, transcription factor binding and chromatin accessibility by DNaseI (see fourth, fifth, and sixth columns). The function of the mapped genes crosschecked with the literature showed evidence of the plurality of risk conditions such as life adversity, unhealthy physical condition and severe physical illness, as well as mental health problems and neurodegenerative diseases, as will be discussed below.

**TABLE 2:** Differentially methylated regions (DMRs) shared by the mothers and their children.

GenId Chr	Cluster Length (N of CpG)	N signif Probes within cluster	Methylation N > C Mother/Child	Genomic coordinates (GRCh37/hg19)	Regulatory Feature Group*	Genomic location (UCSC RefGene)*
*PM20D1* Chr1	14	13	−/+	Start 205818668 N-shore End 205819609 S-shore	CpG island; Promoter; DNaseI hypersensiti-vity	Body TSS1500
*DUSP22* Chr 6	14	12	+/+	Starts 291634 N-shore 293331 S-shore	Promoter_Associated	Body TSS1500
*SLC17A3* Chr 6	8	7	+/+	Start 25882590 S_Shore End 25882328 Island	Unclassified_Cell_type_ specific	Pseudo gene (HIST1H2APS2)
*ZFP57* Chr 6	23	22	−/−	Start 29648161 Ends 29649084	Overlaps enhancer and promoter marks Cell_type_specific	Flanking Tss; Body
*PON1* Chr 7	11	9	−/+	Start 94953653 N-shore End 94954438 S-shore	CpG island; promoter	Body TSS1500
*FGFR2* Chr 10	24	12	−/−	Start 123355239 N-shore End 123356514 N-shore	Overlaps enhancer marks; TF binding; DNaseI hypersensitivity mark	TSS1500; 5′UTR
*WNK4* Chr 17	14	14	+/+	Start 40935998 CpG Island End 40937908 S-shore	CpG island; DNaseI hypersensitivity mark	Body
*URKC* Chr 19	14	13	+/+	Start 557741934 N-shore End 57742444 Island	—	TSS1500 5′UTR; 1stExon
*ZADH2* Chr 18	12	11	−/−	Start; 72916012 N_Shore End 72917390 S_Shore	Promoter_Associated_Cell type_specific; CpG island; DNaseI hypersensitivity mark	Body Body; 5′UTR; 1stExon

Note: (*) Information presented in “Regulatory Feature” and “Genomic location with respect to assigned gene (UCSC_ RefGene)” columns was retrieved from Illumina Manifest (https://we.tl/t-23Rrw2Lr5l) and completed using annotations from UCSC Genome Browser on Human (Illumina Inc., san diego, United States) and HaploReg V4.1 (Ward and Kellis, 2012).

**FIGURE 2:**
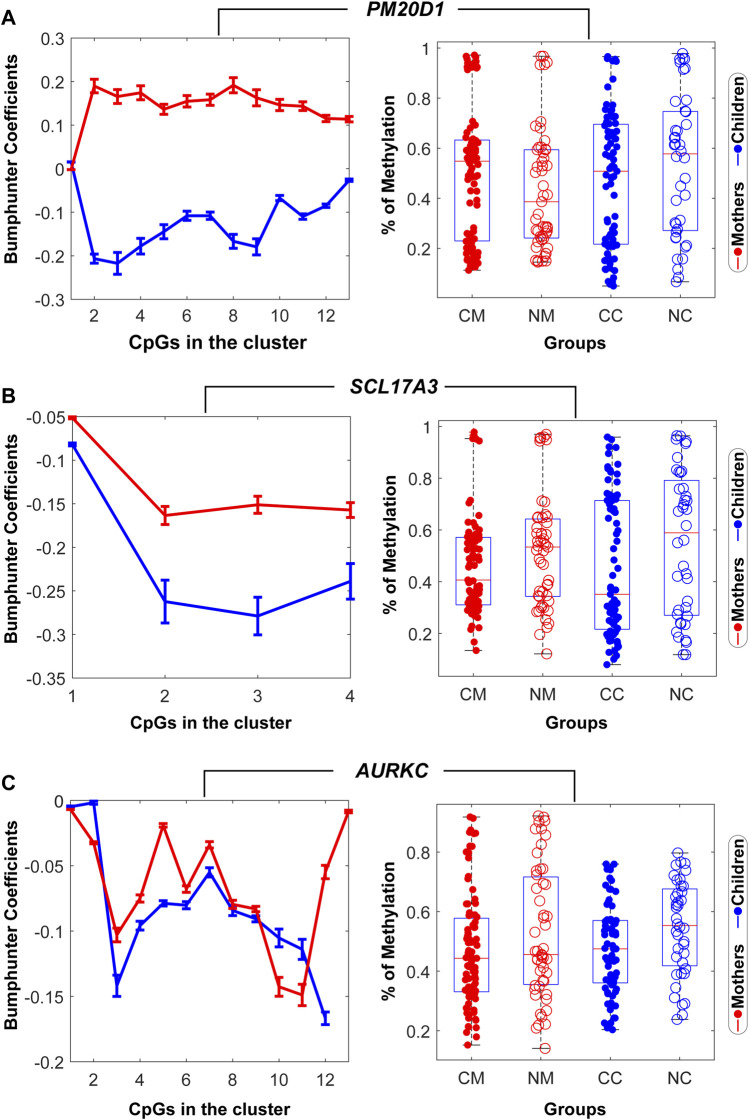
Mother-children methylation patterns in the genes *PM20D1* in **(A)** *, SLC17A3* in **(B)**, and *AURKC* in **(C)**. Left column: Bumphunter coefficients for the group effect (neglect vs. control) across all the CpGs in the cluster for the mother’s data (red) and the child’s data (blue). Right column: Group differences in the mother’s mean methylation (left) and children’s mean methylation (right). CM: control mothers, NM: neglect mothers, CC: control children, NC: neglected children. The residual values of the regression after correcting for the effect of nuisance variables (epithelial cells, maternal age and level of education in the mother data, and child age and maternal age at child’s birth in the child data) were used for this figure.

Three DMRs related to three genes that illustrate the variety of significant patterns in methylation found in mothers and children are presented in Figure 2 (A) *PM20D1*, (B) *SLC17A3,* and (C) *AURKC.* These genes have been found to be associated with life adversity, maltreatment included, in the literature (Suderman et al., 2014: Martins et al., 2021). Bumphunter coefficients for the group effect (neglect vs. control) across all the CpGs in the cluster for the mother data (red) and the child data (blue) followed different patterns in *PM20D1* and similar patterns in *SLC17A3* and *AURKC*. The boxplot of mother and child data showed hypomethylation in the neglect mother and hypermethylation in the neglect child (*PM20D1*), and hypermethylation in both neglect mother and child groups (*SLC17A3* and *AURKC*).

### 4.2 Relationship of the overlapping differentially methylated regions with adverse life events

For the second aim, we studied the relationship between the methylation levels in the DMRs related to the neglect condition with our critical variable measured through the Stressful and Risky Events Inventory (SREI), an index of chronic stress. The resulting global SREI score integrates the number of adverse events and their emotional intensity. Mothers in the neglect group significantly reported higher emotional intensity of adverse life events, M = 16.76, SD = 8.65, than control mothers, M = 11.59, SD = 7.70; *t* (136) = 3.63; *p* < 0.0001, δ = 0.64. Once we had included the effect of other covariates in the Bumhunter design matrix to control them, a negative and significant correlation (*p* < 0.01) was found between the methylation levels and the SREI score for mothers in *PON1*, *FGR2,* and *WNK4*, and also for *ZFP57* for child data. As commented above, for genes PON1, FGR2, and ZFP57, the Control group showed higher methylation levels than the Neglect group. When stress was introduced as a variable to correlate with methylation level, it was found that the higher the stress, the lower the methylation level in both groups. On the contrary, for *WNK4*, the Neglect group showed a higher methylation level than the Control group. The stress was also negatively correlated with *WNK4* methylation for both Neglect and Control subjects. Note that due to the observational nature of our study, causality cannot be attributed here.

## 5 Discussion

This study examined the potential biologically embedding of adverse life experiences through DNA methylation modifications related to neglectful caregiving and whether their neglected children share these epigenetic changes. We also investigated the extent to which shared methylation modifications are related to life adversity exposure, an index of chronic stress reported by mothers, validating this new epigenetic signature of neglectful caregiving beyond the behavioral characterization provided by social services. While life adversity was known to affect DNA methylation in children and adults (Ward and Kellis, 2012), this is the first study on the mother-child epigenetic intersection of these alterations in the context of neglectful caregiving and their potential intergenerational epigenetic modifications. Furthermore, our study overcomes a critical limitation of the literature by reporting the location of individual CpG sites not consistently identified in other studies, which would facilitate further meta-analytic approaches (Parade et al., 2021). The results showed that mothers with neglectful behavior and the neglected children share a differential DNA methylation pattern with respect to mothers and children who are not involved in neglectful caregiving. Specifically, the 69 DMRs in mothers with neglectful behavior and the 42 DMRs in neglected children have in common nine DMRs. Although parents and children share similar exposure to adversity, as evidenced by behavioral studies (Schickedanz et al., 2021), more DMRs are expected to distinguish mothers than children from their respective non-neglectful controls, explained by the increased likelihood of these mothers having an unhealthy lifestyle (Strathearn et al., 2020) and mental health problems (Nelson et al., 2020). This maternal vulnerability may induce a more heterogeneous epigenomic landscape relative to their controls, as these risk factors are also found to affect their epigenetic age (Herrero-Roldan et al., 2021b).

The common set of DMRs and associated mapped genes could be understood as a particular imprint of the neglect setting, since it reflects the differentially methylated regions with respect to those in the mother and child control groups. Noteworthy is the plurality of risky physical and mental health conditions associated with the neglect epigenetic marks. However, this would be expected from the long-lasting severe consequences of exposure to adverse life events for both the mother and the neglected child (Felitti, 2009; Jaffee, 2017; Nelson et al., 2020; Strathearn et al., 2020). Accordingly, methylation in those DMRs areas could be a potential mechanism linking life adversity experiences and later physical and mental health problems (Nestler et al., 2009; Zhang and Meaney, 2010).

The genes mapped in this study have been previously related to early risk conditions traditionally associated with the neglectful setting or with the physical and mental health consequences of early abusive or stressful conditions. Among the genes related to adverse childhood conditions: *PM20D1* was related to both child abuse (Suderman et al., 2014; Benson et al., 2019), *SLC17A3* also to childhood abuse (Suderman et al., 2014) and *AURKC* to early life adversity (Martins et al., 2021). Accordingly, these conditions of early adversity typically match the elevated frequency of the same difficulties in both mother and child in the neglect condition (Petersen et al., 2014; Teicher and Samson, 2016). Interestingly, none of the genes (*NR3C1*, *NR3C2*, *HTR3A*, *SLC6A4*, *OXTR*, and *FKBP5*) previously shown to be differentially methylated in children and adults exposed to maltreatment turned out to be significant in our epigenome-wide analysis (Kertes et al., 2016; Parade et al., 2021). This indicates the involvement of other epigenetic mechanisms when considering shared mother-child genes in neglectful contexts. Studies with target genes found significant mother-child correlation of stress-related genes in a single gene *FKBP5*, in Holocaust survivors (Yehuda et al., 2016) and in *5HTT* and *NR3C1* genes in a general population sample (Van Aswegen et al., 2021). In contrast, differential methylation of the *OXTR* and *IGR* oxytocin-related regions was only observed in the mothers exposed to perinatal depression compared to non-affected mothers, but differential methylation on those genes was not found in their children (King et al., 2017). Similarly, differential methylation in *FKBP5* and *NR3C1* was only observed in mothers with childhood maltreatment concerning mothers without maltreatment experience, but not in their newborns (Ramo-Fernández et al., 2019).

In our epigenetic marks of neglect, genes were also related to nutritional and metabolic alterations and cancer and cardiovascular diseases. *FGFR2* associated with obesity and weight at birth (Haworth et al., 2014; Tian et al., 2018; Carboni et al., 2020) and with adult obesity (Wu et al., 2014); *DUSP22* related to diabetes and obesity (Rizzo et al., 2020), and *ZFP57* related to nutrition/neonatal diabetes (Boonen et al., 2013). In consonance, the neglectful condition has been associated with having a preterm birth (Sulaiman et al., 2021) with lower birth weight (Strathearn et al., 2001) and with developing obesity over the life course (Danese and Tan, 2014; Strathearn et al., 2020). *ZFP57* (Chen et al., 2019), FGFR2 (Xi et al., 2014), and WNK4 (Fackler et al., 2011), have been related to breast cancer, and *WNK4* also with hypertension (Mu et al., 2011; Ching et al., 2021) associated with suffering coronary heart disease (Deschênes et al., 2021), conditions that are common among the long-term consequences of the abuse and neglect (Strathearn et al., 2020).

Psychopathologies are also represented in the set of DMR genes. *PM20D1* (Ensink et al., 2021), *DUSP22* (Rutten et al., 2018), and *ZFP57* (Nöthling et al., 2018; Rutten et al., 2018; Vinkers et al., 2021) were related to post-traumatic stress disorder. *DUSP22* was also related to schizophrenia (Boks et al., 2018), ZFP57 (Ladd-Acosta et al., 2014) with autism, and *FGFR2* (Carboni et al., 2020) with depression. Maternal and child vulnerability to psychopathologies is one of the prominent features of the neglect phenotype due to their high exposure to early social stress (Dias et al., 2017; Conway et al., 2018). Particularly, maternal depression has a high diagnostic value of early child neglect, among other potential indicators (Herrero-Roldán et al., 2021a).

Finally, the epigenetic marks of neglect also contain genes related to neurological problems. *PON1* was associated with child neurobehavioral development (Eskenazi et al., 2010; Holland et al., 2015), and with Parkinson (Mota et al., 2019); *PM20D1* as neuroprotector of Alzheimer (Sanchez-Mut et al., 2018; Li et al., 2021), whereas *DUSP22* (Sanchez-Mut et al., 2018), and ZADH2 (Madrid et al., 2018; Kaur et al., 2022) are considered as a risk condition of Alzheimer. The neglect condition is associated with early neurobiological delays known to be the basis of attentional, emotional, and cognitive problems (Strathearn et al., 2020). Early life adversity was also related to neurodegenerative diseases, such as Alzheimer (Corney et al., 2021). In sum, these findings suggest that epigenetic mechanisms could link the risky health conditions associated with the neglect phenotype in the behavioral studies and the characterization of the disorders associated with the genes reported in the epigenetic field.

Among the set of nine DMR genes, we show in Figure 2 the epigenetic profile of three selected genes that have been previously associated with early adversity and social stress: *PM20D1* (Suderman et al., 2014; Benson et al., 2019); *SLC17A3* (Suderman et al., 2014); and *AURKC* (Martins et al., 2021). We found decreased *PM20D1* methylation and increased *SLC17A3* and *AURKC* methylations in the neglect mothers, and increased *PM20D1*, *SLC17A3,* and *AURKC* methylations in the neglect children, as compared to controls. As noticed in a recent review (Parade et al., 2021), the inconsistent direction of the effects in the same genes is frequently found when reporting results in children and adults under adverse childhood conditions. Although a plausible explanation for this may be the changes in gene expression patterns during life, more longitudinal research is needed to understand how early adversity at each developmental epoch may be associated with differences in epigenetic marks.

Our findings related to the second goal indicate that the epigenetic signature of neglect is sensitive to the maternal exposure chronic stress from adverse life events and potentially translates or extends enduring negative effects to the next generation, at least during childhood. Methylation for *PON1* (associated with neurobiological development, neurodegenerative disease), *FGFR2* (associated with obesity and depression), and *WNK4* (associated with hypertension and obesity) in the mother data, and *ZFP57* (associated with post-traumatic stress disorder) in the child data were correlated to maternal exposure to adverse life events. Behavioral studies have shown that the more adverse life events experienced, the higher the risk for overall physical and mental health (Crouch et al., 2020). The link could be through experiencing chronic stress, thereby increasing the individual’s engagement in health risk behaviors and the likelihood of developing chronic health conditions (Strathearn et al., 2020). This link is most likely to characterize the case of mothers in the neglect group exposed to chronic stress for whom methylation may have detrimental effects on adult development. It could be the case that exposure to neglect in young children can trigger a transient methylation adaptation to acute stress, but also with some potential long-lasting negative effects in line with the experience-dependent adaptation hypothesis (Teicher and Samson, 2016). More longitudinal epigenetic evidence is needed to test the hypothesis.

Behavioral evidence had firmly supported the intergenerational transmission of child abuse and neglect (Bartlett et al., 2017). Our findings of the epigenetic sharing of mother-child genes in methylated regions support the biological explanatory factor for this transmission. Being more precise in our interpretation, the joint signature of methylation between mothers and children in neglect contexts could potentially be explained in three ways. The first is that it is acquired after birth through similar exposure to external environmental and social stressors throughout the mother’s and child’s life. The second is that it is received “biologically” before birth, either through epigenetic (with or without genetic) changes in the mother that are passed on to her developing offspring through the offspring’s fetal germ cells or through the mother’s exposure to risk factors during pregnancy, with both paths able to work simultaneously (Breton et al., 2021). Offspring methylation via germ cells, affected by maternal adverse exposure to trauma or adverse events prior to conception, has received some support (Kertes et al., 2016). Nevertheless, evidence has only been obtained from animal research and even that is limited to date (Scorza et al., 2019). Finally, there is also a third way of transmission after conception due to the external influence of the environment on shared epigenetic alterations. In the postpartum and later periods, neglectful caregiving is characterized by maternal stress that often translates into the poor quality of the immediate mother-child environment and subsequent infant distress (Mulder et al., 2018). As animal research has shown, receiving less care when the pups are exposed to restricted licking and grooming, is a source of stress and suffering that also changes methylation patterns (i.e., Meaney, 2001). Neurological evidence in neglectful caregiving has shown that lower maternal abilities exhibited in close human mother-infant interactions indexed by poor dyadic emotional availability are associated with maternal brain volume reductions in the white matter tract (inferior longitudinal fasciculus). Such tract is involved in the processing of emotional faces (Rodrigo et al., 2016), as well as with volumetric differences in gray and white matter in empathy-related regions (Rodrigo et al., 2019). Thus, it could be possible that a sequence and combination of adverse life events, epigenetic changes and subsequent neurological and behavioral changes may shape the well-known characteristics of the neglectful context.

Despite being the only study to our knowledge with a well-characterized mother-child neglect condition involved in an epigenetic study, our study has several limitations. A large dyadic sample could not be analyzed due to the extreme difficulties of collecting more neglect dyads that met our strict requirements for neglect. The sample size of our study is around the median of epigenetic studies for child maltreatment conducted on separate samples of children and adults (Parade et al., 2021). In addition, significant results were obtained using nuisance covariates and conservative statistical thresholds. Our epigenetic measurements provide inference-only information regarding the actual mechanism in the effector cells in the brain. However, our observation that epigenetic signature is conserved at certain regulatory regions in negligent mothers (vs. controls) suggests that the environmental variables exert their effect through coherent mechanisms involving at least some of these genes. Additional explanatory data of the biological transmission, such as genetic variants (Single Nucleotide Polymorphism) affecting the differentially methylated CpGs, were not assessed due to a lack of sample genotyping. Future studies will consider SNP genotyping, which will be integrated into the analyses. Importantly, methylation was derived from salivary samples, which has its own unique methylation profile compared to other tissues (Smith et al., 2015; Middleton et al., 2022). Additional tissues such as blood and brain should also be examined due potential tissue-specific DNA methylation associations (Smith et al., 2015). The use of retrospectively reported measures for adverse life events relying on the recall can always be a source of bias, even though negative events enhanced by emotional impact are less prone to memory bias than positive events (Earles et al., 2016). Finally, besides the mothers’ experience of adverse life events, it would have been desirable to measure the maternal exposure to childhood maltreatment and other lifestyle factors, such as dietary, drug consumption, or physical diseases.

## 6 Conclusion

This study provides the first evidence of a shared epigenetic signature associated with the neglect phenotype involving the mother who performs the neglectful behavior and the child who suffers from it. The mother-child epigenetic mark of neglect embodies a crucial associative pathway, not necessarily causative, that sheds light on the intergenerational problem of maltreatment. Relationships between the shared DMRs and maternal chronic stress related to adverse life events may help explain how maternal adversity extends its biological echo to offspring by increasing the risk of subsequent cognitive and emotional dysfunctions and, eventually, later vulnerability to physical and mental health problems. A further step performed in this study is that this common biological pathway may also help explain how mothers’ chronic stress in the neglect group was linked to their propensity for suffering unhealthy physical conditions, mental health problems and neurodegenerative diseases in their life. Additional studies exploring shared DNA mother-child methylation may also reveal whether the epigenetic mark of neglect is partially or fully present in other types of child maltreatment, such as physical and sexual abuse. In sum, understanding how mother-child altered methylation passes on to the potential unhealthy development of the offspring would expand the possibilities for early diagnosis of the neglect condition in both the mother and child and for targeted interventions to prevent and ameliorate the negative impact of maternal adversities in mother-child caregiving contexts and subsequent health problems.

## Data Availability

The mother and child Beta values corresponding to the Differential Methylation Regions used for the original contributions presented in the study are publicly available at https://doi.org/10.5061/dryad.wwpzgmsn4. The full disclosure of the raw sequencing data of mothers and their minors is subject to confidentiality restrictions of the Canary Islands Child Protection Services. Requests to access the raw data should be directed to the corresponding author.
